# Optimization and scale-up production of Zika virus ΔNS1 in *Escherichia coli*: application of Response Surface Methodology

**DOI:** 10.1186/s13568-019-0926-y

**Published:** 2019-12-31

**Authors:** Alex Issamu Kanno, Luciana Cezar de Cerqueira Leite, Lennon Ramos Pereira, Mônica Josiane Rodrigues de Jesus, Robert Andreata-Santos, Rúbens Prince dos Santos Alves, Edison Luiz Durigon, Luís Carlos de Souza Ferreira, Viviane Maimoni Gonçalves

**Affiliations:** 1 Laboratório de Desenvolvimento de Vacinas, Instituto Butantan, Av Vital Brasil, 1500, São Paulo, SP 05503-900 Brazil; 2 Laboratório de Desenvolvimento de Vacinas, Instituto de Ciências Biomédicas, Universidade de São Paulo, São Paulo, SP Brazil; 3 Laboratório de Virologia, Instituto de Ciências Biomédicas, Universidade de São Paulo, São Paulo, SP Brazil

**Keywords:** Zika NS1, Serological diagnosis, *E. coli*, Heterologous protein production, Soluble expression, Response Surface Methodology

## Abstract

Diagnosing Zika virus (ZIKV) infections has been challenging due to the cross-reactivity of induced antibodies with other flavivirus. The concomitant occurrence of ZIKV and Dengue virus (DENV) in endemic regions requires diagnostic tools with the ability to distinguish these two viral infections. Recent studies demonstrated that immunoassays using the C-terminal fragment of ZIKV NS1 antigen (ΔNS1) can be used to discriminate ZIKV from DENV infections. In order to be used in serological tests, the expression/solubility of ΔNS1 and growth of recombinant *E. coli* strain were optimized by Response Surface Methodology. Temperature, time and IPTG concentration were evaluated. According to the model, the best condition determined in small scale cultures was 21 °C for 20 h with 0.7 mM of IPTG, which predicted 7.5 g/L of biomass and 962 mg/L of ΔNS1. These conditions were validated and used in a 6-L batch in the bioreactor, which produced 6.4 g/L of biomass and 500 mg/L of ΔNS1 in 12 h of induction. The serological ELISA test performed with purified ΔNS1 showed low cross-reactivity with antibodies from DENV-infected human subjects. Denaturation of ΔNS1 decreased the detection of anti-ZIKV antibodies, thus indicating the contribution of conformational epitopes and confirming the importance of properly folded ΔNS1 for the specificity of the serological analyses. Obtaining high yields of soluble ΔNS1 supports the viability of an effective serologic diagnostic test capable of differentiating ZIKV from other flavivirus infections.

## Introduction

Zika virus (ZIKV) is a flavivirus transmitted primarily by *Aedes aegypti* mosquitoes. Many cases of microcephaly and other congenital malformations were reported following ZIKV infections during pregnancy (Franca et al. 2016). Most cases are asymptomatic, however, infection of both children and adults can lead to serious neurologic complications, such as Guillain-Barré syndrome or neuropathy (WHO 2016). The ZIKV infections are normally diagnosed by molecular tests designed to detect viral RNA in the blood or saliva, but the short bloodstream viral detection window limits its utilization. Furthermore, another concern lies in the molecular tests reliability, since recent reports showed 73% of suboptimal sensitivity or specificity among 15 Brazilian laboratories (Fischer et al. 2018) and similar results among European laboratories, which highlights the challenging aspect of the diagnosis.

On the other hand, serological tests do not have the disadvantage of limited window of detection. IgM and IgG antibodies can be detected for months or even years following the ZIKV infection (Chua et al. 2017; Paz-Bailey et al. 2018). Moreover, serological tests also have the advantage of being easily implemented due to lower costs and technical requirements. Since the ZIKV outbreak in the Northeast of Brazil, there was a major effort towards the development of a reliable serological test. For other flavivirus, particularly DENV, the laboratory diagnostic was mostly based on the detection of antibodies against the non-structural protein 1, NS1 (Kikuti et al. 2018; Stettler et al. 2016). Previous work indicated that serological assays based on NS1 can be used to discriminate ZIKV and DENV infections (Balmaseda et al. 2017; Bosch et al. 2017; Stettler et al. 2016). However, ZIKV and DENV proteins share high sequence identity resulting in the cross-reactivity of antibodies generated after the infection (Balmaseda et al. 2017; Fernanda Estofolete et al. 2016; Granger et al. 2017; Priyamvada et al. 2017). In spite of significant progress made in the last years in our understanding of ZIKV, the improvement of diagnostic assays is still needed (Kikuti et al. 2018; Theel and Hata 2018).

We have previously produced a recombinant ZIKV NS1 protein initially as insoluble inclusion bodies, which required the use of high hydrostatic pressure in order to refold it. Even though refolded NS1 from ZIKV and DENV preserved the antigenic properties (Amorim et al. 2010; Rosa da Silva et al. 2018), the refolding is generally avoided since it requires more steps of purification and increases the overall cost of the process (Vallejo and Rinas 2004; Yang et al. 2011). In recent studies, we have produced a recombinant protein derived from the ZIKV NS1 protein (ΔNS1) (Caires-Junior et al. 2018; Kam et al. 2017; Oliveira et al. 2016). To be used as a diagnostic test, ΔNS1 needs to be produced at high yields in its soluble form.

*Escherichia coli* is one of the most used hosts to produce recombinant proteins due to its well characterized genetics and the abundant number of strains and vectors (Baneyx 1999; Baneyx and Mujacic 2004; Rosano and Ceccarelli 2014). Furthermore, other variables such as medium components, additives and culture conditions, allows a series of combinations between these factors and increase the challenge in determining which factors are important in order to obtain high yields of properly folded recombinant proteins. Even if we restrict to the most important factors affecting the production of recombinant proteins, testing one-factor-at-a-time will require many experiments. The Response Surface Methodology is a statistical tool used to deal with several variables that affect a particular response, including the production of recombinant proteins (Larentis et al. 2011; Marthos et al. 2015; Montgomery 2008; Papaneophytou and Kontopidis 2012). Using *E. coli* BL21 (DE3) as host to express 104 ORFs from different organisms in standard conditions (induction at 37 °C with 0.5 mM IPTG for 3 h), Abergel et al. (2003) demonstrated that 89 exhibited detectable expression and 54 were obtained in soluble form (52%). On the other hand, using a statistical design to evaluate *E. coli* strains, culture media and different temperatures, out of the 94 ORFs tested, 93 showed detectable expression and 68 were soluble. This represents 72% of the proteins obtained in the soluble form, thus demonstrating that optimization of culture conditions through statistical designs can improve the solubility of the recombinant proteins.

The goal of this work was to increase the yield of ΔNS1 by improving the growth of the *E. coli* host and solubility of ΔNS1. The present study applied Response Surface Methodology (RSM) to evaluate different culturing conditions. We validated the model, scaled-up the production and evaluated the performance of ΔNS1 in serological tests to discriminate between the ZIKV and DENV infections in mouse and human.

## Materials and methods

### Plasmids construction and bacterial strains

Recombinant plasmids (GenScript, USA), derived from pET-28a, encoding the full sequence of DENV-2 strain NGC (GenBank reference number M29095) and ZIKV NS1 based on the Brazilian strain (GenBank reference number ALU33341) were introduced into chemically competent *E. coli* BL21-Codon Plus (DE3)-RIL strain (Stratagene, USA). The plasmid encoding the ZIKV ΔNS1 carries the genetic sequence corresponding to the last 100 amino acids of the C-terminal region of ZIKV NS1 (patent application number: BR 10 2016 011318 0). The plasmid encoding DENV-2 NS1 was previously reported (Amorim et al. 2010). Recombinant expression plasmids encoding the ΔNS1 were introduced in *E. coli* Arctic Express (DE3) (Agilent) and BL21 (DE3) (Invitrogen) strains.

### Evaluation of culture media for the expression of ΔNS1

The expression of the full DENV-2 and ZIKV NS1 proteins was performed according to protocols described previously (Amorim et al. 2010). To produce ZIKV ΔNS1, the clones were grown in LB (Yeast extract 5 g/L, Tryptone 10 g/L, NaCl 10 g/L), TB (Yeast extract 24 g/L, Tryptone 12 g/L, glycerol 4 mL/L, KH2PO4 2.31 g/L, K_2_HPO_4_ 12.54 g/L) or 2xHKSII medium [Yeast extract 10 g/L, tryptone 20 g/L, acid hydrolyzed casein 4 g/L, salts Mg, K and Ca, and trace metals Fe, Zn, Mn, Cu, Co, B, Mo and I, as described by Jensen and Carlsen (1990)], containing kanamycin (20 µg/mL). A cell bank was prepared for each bacterium and the same procedure was performed for every culture. Briefly, a single clone was grown in LB or TB with kanamycin until mid-log phase, centrifuged and cells washed with phosphate-buffered-saline. The cell pellet was resuspended with medium and aliquots stored with 10% glycerol at − 80 °C until use. Before each run, a pre-inoculum was prepared a day before by inoculating an aliquot of the frozen stock in 50 mL of medium kept at 37 °C for 16 h at 200 rpm using a Gyromax 737R incubator (Amerex, USA). 500 mL-Erlenmeyer flasks containing 50 mL of medium were inoculated with the pre-inoculum to an initial optical density (OD) at 600 nm of 0.1 and grown at 37 °C at 250 rpm. When the cultures reached an OD ~ 2.0, they were transferred to 11 °C (Arctic strain) and 16 °C (BL21 DE3 strain) for 30 min without shaking. Cultures were induced with 0.5 mM of IPTG (Aldrich Sigma, USA) and incubated in the same temperature at 250 rpm for 18 h.

### Response Surface Methodology setup

After the selection of the strain and culture medium with the highest ΔNS1 solubility (BL21 DE3 in TB, with the addition of anti-foam polypropylene glycol (PPG) 0.03% (v/v)), a Central Composite Rotatable Design (CCRD) was created with the aid of the Design Expert software 7.0. Based on the literature and our previous data, three factors were selected as independent variables: temperature, time of harvest after induction and inducer concentration, and the design comprised a total of 20 experiments, 14 experimental variations, including 6 axial points with an alpha of 1.68, and 6 centerpoints (Table 1). Before each run, the previously prepared glycerol stocks were inoculated into 500 mL-Erlenmeyer flasks with 50 mL of TB and incubated at 37 °C for 16 h at 200 rpm. The pre-inoculum was used to seed full-baffled TunAir mini flasks (Aldrich Sigma, USA) with 100 mL medium to an initial OD of 0.1 and cultures were grown at 37 °C at 250 rpm. When the OD of the cultures reached approximately 2.0, they transferred to the specified temperatures according to the experimental design and incubated for 30 min without shaking. The cultures were then induced with the indicated amount of IPTG, incubating at the respective temperatures with shaking at constant 250 rpm. The effect of the variables and their interactions were considered statistically significant when *p* < 0.05. Non-significant factors were excluded from the model.

**Table 1:** Independent variables and levels of the experimental design

Independent variable	Level				
	− α^a^	− 1	0	+ 1	+ α^a^
A: Temperature (°C)	11.2	16	23	30	34.8
B: Time (h)	7.9	12	18	24	28.1
C: IPTG (mM)	0.2	0.4	0.7	1.0	1.2

^a^ α = 1.68

### Quantification of biomass production and soluble ΔNS1

After each cultivation the final OD was measured and the equivalent volume of an OD 5.0 was collected (e.g. collect 1 mL of OD 5.0 or 2 mL of OD 2.5). The suspension was washed with lysis buffer (100 mM Tris, 500 mM NaCl, 1 mM PMSF, pH 8.5) and resuspended in a final volume of 0.5 mL. Bacteria were disrupted by sonication using an Ultrasonic Processor GE 100 (3 cycles of 5 min with 1 s pulses at 60 Hz). Soluble and insoluble fractions were separated by centrifugation at 14,000*g*, at 4 °C for 30 min. The protein extracts were separated by SDS-PAGE (15% gels). The gels were stained with Coomassie Blue R-250 for 16 h and unstained using a destaining solution (30% ethanol, 10% acetic acid v/v). To evaluate the production of ΔNS1, the band area corresponding to its predicted mass (~ 18 kDa) was measured by densitometry using ImageJ software. A standard curve of BSA was used to determine the concentration of ΔNS1. Total yield (mg/L) was calculated considering the culture volume collected and the volume applied to the gel. Cell concentration was measured as OD and converted to dry cell weight (DCW). To calculate DCW, 500 mL of culture was taken after the bioreactor cultivation and inactivated using 2% formalin for 16 h. Serial dilutions were made and OD measured for each dilution. Pre-determined volumes of the samples were centrifuged and the cell pellets taken to an oven set to 100 °C. After at least 48 h or until constant weight, cell pellets were weighed and plotted against the OD to establish the correlation of OD vs DCW, resulting in 1 unit of OD to 0.34 g/L of DCW.

### Bioreactor setup

The best condition identified in the shake-flask experiments was carried out in the 6-L bioreactor (BioStat C-Plus, Sartorius) in a batch cultivation using the chosen medium with addition of kanamycin (20 µg/mL) and anti-foam PPG (0.03% v/v). The inoculum of the bioreactor was prepared as follows: 1 L Erlenmeyer flask containing 100 mL of medium was previously cultured overnight and used to inoculate the bioreactor to an initial OD of 0.1. Dissolved oxygen (pO2) was maintained at 30% air saturation and pH at 7.2, controlled by the automatic addition of phosphoric acid 98% or ammonium hydroxide 24%. The culture was maintained at 37 °C until OD reached ~ 2.0, then the temperature was decreased to 21 °C and IPTG was added according to the values determined in the shake-flask experiments. Cell concentration was measured by regular readings after inoculation, and samples were also used to determine the production and solubility of ΔNS1.

### Protein purification

The recombinant ZIKV proteins, as well as the NS1 originally derived from DENV-2 NGC strain, were purified by affinity chromatography according to previously described methodology (Amorim et al. 2010). For ΔNS1 an additional purification step was necessary using size-exclusion chromatography with a previously described protocol (Caires-Junior et al. 2018).

### ELISA

Polystyrene high-binding COSTAR microplates (Corning Inc., New York, EUA) were coated with 200 ng of ZIKV ΔNS1 or equimolar amounts of the ZIKV and DENV NS1 proteins in phosphate-buffered saline (PBS) pH 7.2, overnight at 4 °C, and, then, blocked with 5% skimmed milk and 1% BSA for 2 h at room temperature. The plates were washed three times in PBS-Tween 0.05% (PBST) and human serum samples from ZIKV and/or DENV-infected individuals were serially diluted (log2) in 5% skimmed milk and 0.25% BSA and incubated at room temperature for 1.5 h. The DENV^+^ sera were obtained from eight patients infected with serotypes 1 to 4 (Alves et al. 2016). After a washing cycle, the diluted goat anti-human IgG peroxidase conjugate (Aldrich Sigma, USA; reference code: A0170) was added to the wells and incubated again for 1.5 h. After a final washing step, plates were developed with citrate buffer (pH 5.8) containing 0.4 mg/mL of ortho-phenylenediamine dihydrochloride (OPD) (Aldrich Sigma, USA) and 0.12% H_2_O_2_. The reaction was stopped after 15 min with the addition of 50 µL/well of 2 N H_2_SO_4_. The OD of the reaction was measured at 492 nm in a plate reader (Labsystems Multiskan, Thermo Scientific, USA). For assays using the denatured forms of the proteins, these were previously heated to 100 °C for 10 min and immediately cooled to 0 °C in ice. The proteins were then used to coat the plates as described above. To evaluate the specificity of ZIKV ΔNS1 and DENV NS1 proteins in mice infected with ZIKV and other Flavivirus, the same protocol was used with goat anti-mouse IgG peroxidase conjugate (Aldrich Sigma, USA; reference code: A4416).

### Statistical analysis

Design Expert 7 Software was used to analyze the results of CCRD. The quality of the regression of the model equation was evaluated by the coefficients R^2^, adjusted R^2^ and predicted R^2^. The significance of the factors and interactions was determined by the tabulated and calculated F-value at p = 0.05. The lack-of-fit test was used to evaluate the differences of experimental and pure error. An insignificant lack-of-fit test (p-value > 0.05) states that the model correctly represents the correlation between response and predictors. Adequate precision was used to measure the signal-to-noise ratio. Ratios greater than 4 suggest that the model is adequate in predicting the responses within the space design. The normal probability plot was used to observe the distribution of the residuals. Differences in the antibody levels determined by ELISA were calculated using two-way ANOVA and the Bonferroni test. Differences with p < 0.05 were considered statistically significant.

## Results

### Response Surface Methodology for growth and production of soluble ΔNS1

A preliminary evaluation of strains and culture media was performed to exclude some variables and reduce the number of experiments in the RSM design. Since solubility was an important aspect, we sought to compare the solubility of ΔNS1 produced by *E. coli* Arctic Express (DE3) and BL21 (DE3) strains using LB, TB and 2xHKSII. In the protein extracts of the cold-adapted Arctic strain it was possible to detect soluble ΔNS1 but at very low levels, only detectable by Western blot. Only the BL21 strain cultured and induced in TB showed a distinguishable band of soluble ΔNS1 in Coomassie stained SDS-PAGE (Additional file 1: Figure S1) and therefore were used for further optimization using the statistical design. For the statistical design we used full-baffled shake flasks, which resulted in a much higher final OD (OD ~ 18.0), but also required the addition of anti-foam. Furthermore, in the presence of the anti-foam ΔNS1 showed increased solubility (Additional file 1: Figure S2).

The RSM design, coded independent variables (time, temperature and IPTG), actual and predicted responses (biomass and production of soluble ΔNS1) are summarized in Table 2. Throughout the experiments, biomass varied between 2.02 and 7.76 g/L and ΔNS1 from 5 to 1154 mg/L. The effects of temperature, time of induction and IPTG concentration on biomass and ΔNS1 concentration are shown in Table 3. The time of induction presented a positive significant effect on biomass (p < 0.0001). The interaction between time and temperature presented a negative significant effect on biomass (p < 0.0001) and all quadratic factors were significant and had negative effect on this response. The quadratic effect of the temperature presented the major influence on biomass (the lowest coefficient, − 1.86), which makes sense, as values below or above the optimum temperature range will diminish cell growth (Table 3). For ΔNS1, the effect of the temperature was significant (p < 0.05) and negative, which means that high induction temperatures led to ΔNS1 aggregation into inclusion bodies. The interaction between time and temperature of induction also had a significant negative effect on ΔNS1 solubility (p = 0.01), indicating that the formation of inclusion bodies increased with time and temperature. All quadratic factors presented significant negative effect on ΔNS1 solubility (p < 0.0001) and the square of temperature showed the more pronounced effect (Table 3).

**Table 2:** Results of CCRD used to assess the influence of temperature, time of induction and IPTG concentration on biomass and soluble ΔNS1 production

Run	Independent variables^a^			Responses			
				Biomass (DCW g/L)		ΔNS1 (mg/L)	
	Temp	Time	IPTG	Experimental	Predicted	Experimental	Predicted
1	− 1.68	0	0	2.02	2.30	229	278
2	+ 1	+ 1	− 1	2.81	3.45	52	7
3	0	− 1.68	0	3.33	3.51	344	279
4	− 1	− 1	− 1	2.10	1.92	168	144
5	+ 1.68	0	0	2.51	1.89	5	14
6	+ 1	+ 1	+ 1	3.17	3.45	97	38
7	+ 1	− 1	+ 1	3.92	4.08	292	286
8	− 1	− 1	+ 1	2.17	1.92	131	175
9	0	0	+ 1.68	4.97	5.16	357	386
10	0	0	0	6.78	7.35	802	969
11	− 1	+ 1	+ 1	6.39	6.09	526	463
12	0	0	− 1.68	5.68	5.16	304	334
13	0	0	0	7.65	7.35	1021	969
14	0	0	0	7.10	7.35	837	969
15	0	+ 1.68	0	6.99	6.48	190	313
16	− 1	+ 1	− 1	5.63	6.09	506	432
17	+ 1	− 1	− 1	3.92	4.08	194	254
18	0	0	0	7.76	7.35	1154	969
19	0	0	0	7.10	7.35	917	969
20	0	0	0	7.65	7.35	1095	969

^a^Code values for independent variables, the actual values in Table 1

**Table 3:** Estimated effects for IPTG concentration, temperature and time of induction on biomass and ΔNS1 solubility

Factors	Biomass (DCW g/L)			ΔNS1 (mg/L)		
	Effect	Standard error	p-value	Effect	Standard error	p-value
Mean	7.35	0.21	< 0.0001	969	49	< 0.0001
A: Temp	− 0.12	0.14	0.4032	− 79	32	0.0352
B: Time	0.88	0.14	< 0.0001	10	32	0.7601
C: IPTG	0.00	0.14	0.9975	16	32	0.6377
AB	− 1.20	0.18	< 0.0001	− 134	42	0.0100
AC	− 0.06	0.18	0.7486	20	42	0.6461
BC	0.13	0.18	0.4851	0	42	0.9915
A^2^	− 1.86	0.14	< 0.0001	− 291	31	< 0.0001
B^2^	− 0.83	0.14	< 0.0001	− 238	31	< 0.0001
C^2^	− 0.78	0.14	0.0002	− 215	31	< 0.0001

**Fig. 1:**
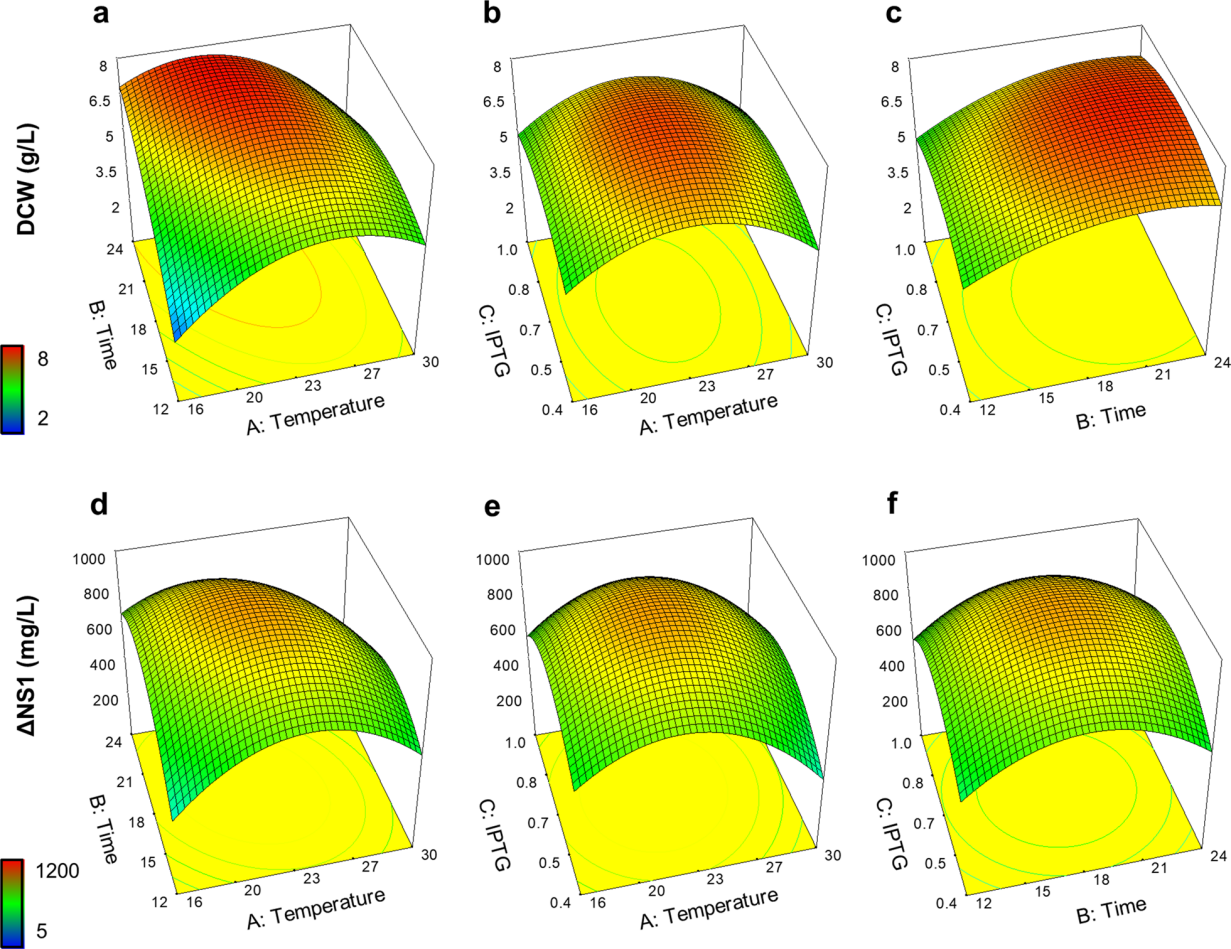
Response surface plots for biomass and production of soluble ΔNS1 as a function of time, temperature of induction and IPTG concentration. The model generated by the CCD for **a**–**c** biomass concentration measured as dry cell weight (DCW g/L) and **d**–**f** production of ΔNS1 the soluble fraction of cell extracts (mg/L). The temperature range was 16–30 °C, time between 12 and 24 h of induction and IPTG concentration between 0.4 and 1.0 mM. Color-coding indicates high (red) and low (blue) responses

The conditions that generated the highest biomass and ΔNS1 concentration in the soluble fraction were observed at the centerpoint, 23 °C for 18 h and 0.7 mM IPTG, which produced an average of 7.34 ± 0.4 DCW g/L and 954 ± 120 mg/L of ΔNS1. The lowest temperature (11.2 °C) produced the lowest biomass concentration, while the highest temperature (34.8 °C) resulted in the lowest amount of soluble ΔNS1. For biomass, the response surface plot for temperature (A) and time (B) showed a rising ridge shape towards longer times and lower temperatures (Fig. 1a). In Fig. 1b we observe the positive effect of mild temperatures and in Fig. 1c the positive effect of longer times on cell growth. In both cases, the influence of IPTG concentration on biomass response is less pronounced than the other two factors (Fig. 1b, c). All surface plots showed less intense effects of independent variables on ΔNS1 production (Fig. 1d–f) than on biomass (Fig. 1a–c). The ANOVA table showed that the empirical model was statistically significant (p < 0.0001), while the lack of fit was insignificant (Table 4). The normal probability plot of the studentized residuals showed normal distribution and residuals were insignificant for both biomass and ΔNS1 responses (Additional file 1: Figure S3).

**Table 4:** Analysis of variance (ANOVA) of the influence of temperature, time and IPTG concentration on biomass and ΔNS1 solubility

Source of variation	Biomass (DCW g/L)^a^					ΔNS1 (mg/L)^b^				
	Sum of squares	df	Mean square	F-calc	p-value	Sum of squares	df	Mean square	F-calc	p-value
Regression	82.49	9	9.17	34.83	< 0.0001	2.5 × 10^6^	9	2.8 × 10^5^	19.52	< 0.0001
Residuals	2.63	10	0.26			1.4 × 10^5^	10	14,251		
Lack of fit	1.83	5	0.37	2.30	0.1913	4.2 × 10^4^	5	8335	0.31	0.8228
Pure error Total	0.80	5	0.16			1.0 × 10^5^	5	20,168		

^a^Model: 7.35 + 0.88*B − 1.20*AB − 1.86*A^2^ − 0.83*B^2^ − 0.78*C^2^, R^2^ = 0.97; Adj. R^2^ = 0.95; Pred. R^2^ = 0.92. Adeq. precision = 21.9. F-tab = 2.96

^b^Model: 969 − 79*A − 134*AB − 291*A^2^ − 238*B^2^ − 215*C^2^, R^2^ = 0.94; Adj. R^2^ = 0.92; Pred. R^2^ = 0.90. Adeq. precision = 16.9. F-tab = 2.96

### Predicted versus actual DCW and ΔNS1 production and model validation

**Fig. 2:**
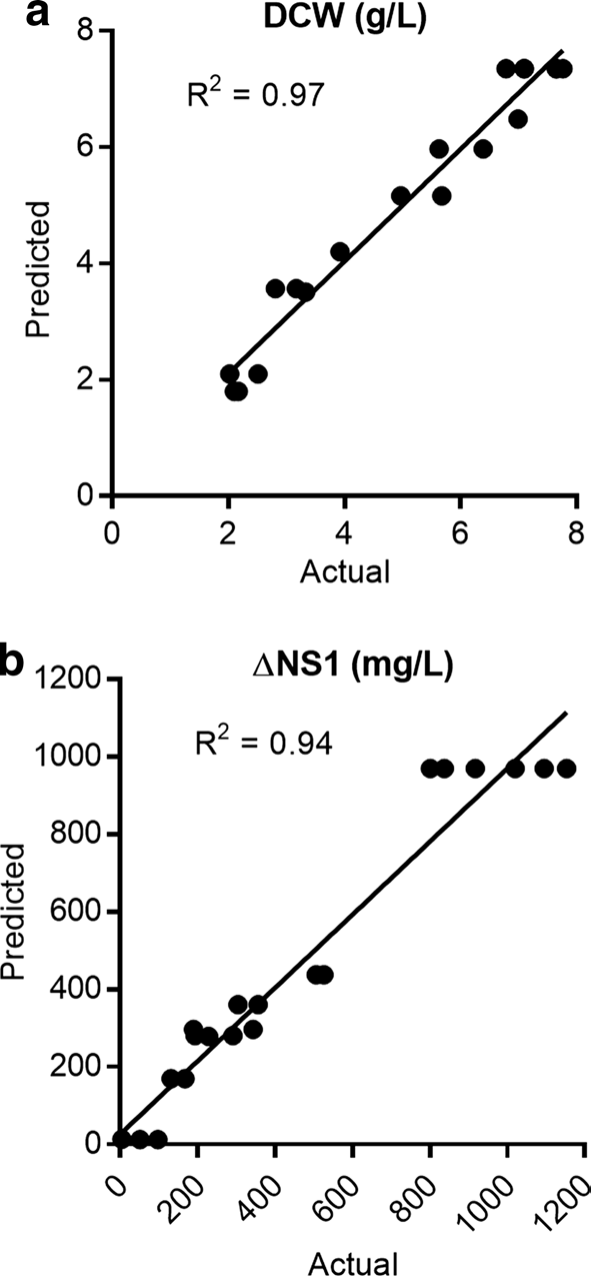
Predicted vs actual plots for biomass and ΔNS1. Linear regression plot for the predicted and actual responses for **a** biomass formation (g/L DCW) and **b** ΔNS1 (mg/L) obtained in soluble fraction of cell extracts

The experimental data for DCW and ΔNS1 production were compared to the values predicted by the model (Fig. 2a, b). Both responses showed good correlation between the actual and the predicted values (R^2^ = 0.97 and 0.94 for DCW and ΔNS1, respectively). According to the model, the highest biomass concentration (7.7 g/L) would be obtained at 21.5 °C, 22 h, and the highest yield of ΔNS1 in the soluble fraction (975 mg/L) at 22 °C, 18 h. The best solution for both responses would be 21 °C for 20 h. This condition predicted 7.5 g/L DCW and 962 mg/L of ΔNS1. To validate the model generated by the RSM design we performed three independent runs at predicted optimal condition for both dependent variables. The results we obtained were very close to those predicted by the model. On average 7.25 ± 0.25 g/L DCW and 1022 ± 29 mg/L of ΔNS1 were produced (Table 5).

**Table 5:** Biomass and soluble ΔNS1 production of the validation experiments

	Biomass (DCW g/L)	ΔNS1 (mg/L)
Predicted	7.50	963
Experiment 1	7.54	1033
Experiment 2	7.10	989
Experiment 3	7.10	1043
Experiment average	7.25 ± 0.25	1022 ± 29

### Bioreactor

**Fig. 3:**
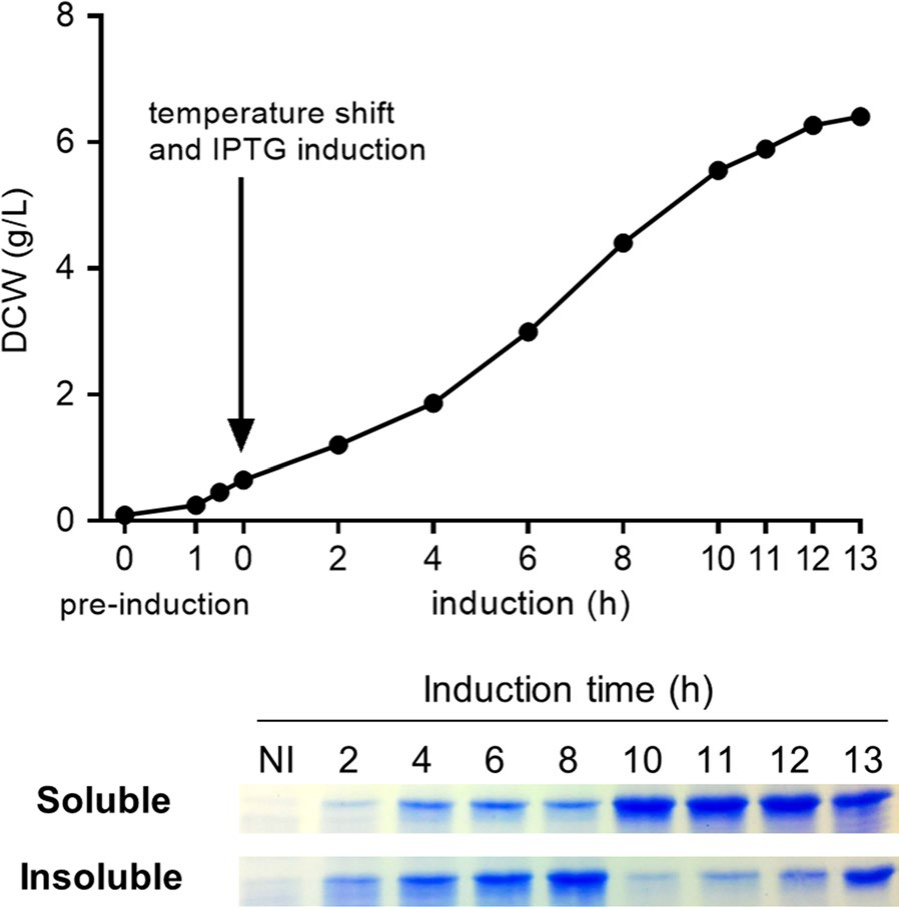
Solubility of ΔNS1 during the induction in the bioreactor. After inoculation to achieve OD 0.1, culture was maintained at 37 °C until OD ~ 2.0 (pre-induction). Temperature was shifted to 21 °C and protein production induced with 0.7 mM IPTG. To determine solubility and biomass concentration, aliquots were taken at regular intervals after inoculation. After lysis, soluble and insoluble protein extracts were separated by SDS-PAGE and stained by Coomassie Blue. NI = non-induced. OD converted to biomass (g/L) according to the relation 1.0 OD = 0.34 g/L

Based on the culture conditions established in the shaken-flask experiments, we scaled-up ΔNS1 production to a 6-L batch. In the bioreactor, induction was performed at 21 °C and 0.7 mM IPTG, while regular readings provided information on growth and solubility of ΔNS1 across time. Biomass reached its maximum value (DCW 5.5–6.4 g/L) at 10–12 h after induction. Soluble and insoluble ΔNS1 were observed as soon as 2 h after the induction. The maximum of soluble protein was observed after 10–12 h of induction. Further cultivation greatly affected the solubility of ΔNS1, which starts to decrease and aggregates in inclusion bodies (Fig. 3). At the same time, specific growth rate and plasmid stability also decreases from 0.24 to 0.1/h and from 99 to 56% of antibiotic resistant colonies, respectively, suggesting that an overall stress may be occurring after 10–12 h of induction (data not shown). For controlling the pH, a total of 37 mL phosphoric acid and 1 mL ammonium hydroxide were added during the process.

### Validation of the specificity of the antigen produced

**Fig. 4:**
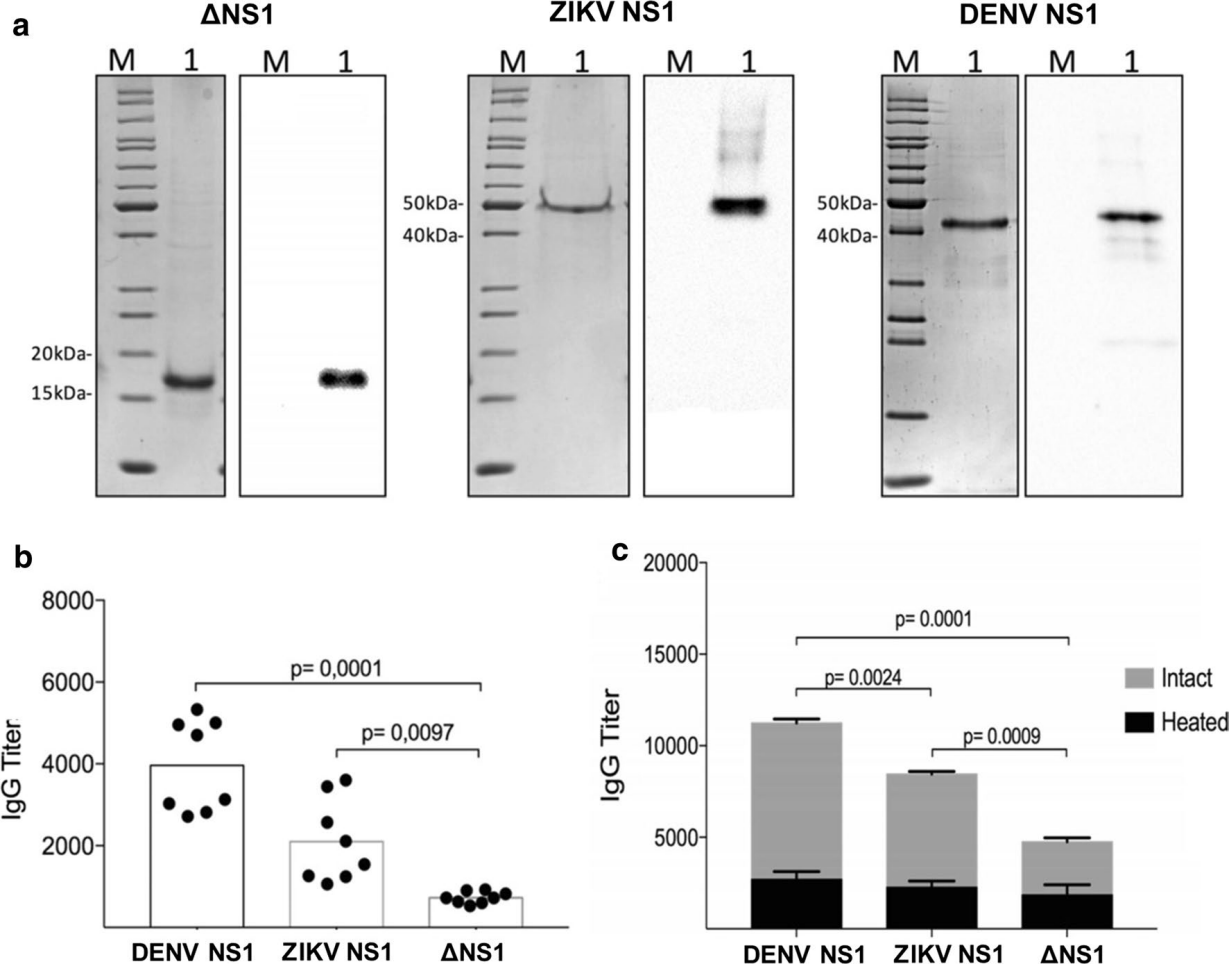
Antigenicity and specificity of the recombinant protein ΔNS1. **a** Coomassie Blue staining (left panels) and Western blots (right panels) obtained with 1 µg of purified ΔNS1 or full length Brazilian ZIKV NS1 and DENV2 NS1 (strain NGC). Western blots were probed with a mAb anti-His-Tag (ZIKV proteins) or anti-DENV2-NS1. **b** Reaction of human immune sera from eight DENV-infected subjects (serotypes 1 to 4) with DENV NS1, ZIKV NS1 and ΔNS1 in ELISA. **c** Reaction of ZIKV^+^ DENV^+^ human serum sample with intact and heat denatured DENV NS1, ZIKV NS1 and ΔNS1. Statistical significance was assessed by two-way ANOVA and the Bonferroni test

After establishing the best expression conditions, the next step was to determine the recombinant protein specificity for the detection of virus-specific antibodies raised among ZIKV-infected subjects, particularly those previously infected with DENV. We produced and purified ΔNS1 as well as the full length NS1 of the Brazilian ZIKV strain and the NS1 derived from type 2 DENV (NGC strain) (Fig. 4a). To test the specificity of the ∆NS1 in serological assays, we standardized an ELISA with the above-mentioned proteins and probed them with monovalent ascitic fluids from mice infected with DENV2, ZIKV, YFV or CHIKV (Additional file 1: Figure S4a). The results showed reduced (DENV) or no (YFV and CHIKV) cross-reactivity with ΔNS1. Similarly, there was low cross-reactivity of ∆NS1 with sera from mice infected with DENV1, DENV3 and DENV4 (Additional file 1: Figure S4b). In contrast, antibodies of mice infected with ZIKV strongly reacted with ΔNS1, which indicated that the protein preserved the targeted epitopes under the established production conditions. Antigen validation was also assessed with serum samples collected from eight patients infected with DENV serotypes 1, 2, 3 and 4 before the entry of ZIKV in South America (Fig. 4b) or double-positive for ZIKV/DENV (Fig. 4c). The DENV^+^ sera for all four serotypes exhibited significantly lower reactivity with ΔNS1 as compared to the full length ZIKV NS1 (50% reduction) and to the DENV NS1 (75% reduction) (Fig. 4b), indicating its reduced cross-reactivity. In a serum sample collected from a subject previously exposed to DENV and ZIKV, the ΔNS1 antigen showed significant reactivity. ZIKV NS1 and DENV NS1 reactivities were significantly increased as compared to that observed for the ΔNS1 antigen, as expected (Fig. 4c). Notably, heat denaturation of the antigens drastically reduced the reactivity of the tested serum antibodies (Fig. 4c).

## Discussion

The initial results with ΔNS1 indicated that it would be a promising candidate for the differential serological diagnosis of ZIKV infections in the presence of DENV and CHIKV. However, it is necessary to obtain the protein in high yields and in soluble form. Previous observations showed that insoluble NS1 in the form of inclusion bodies could be renatured by a high-pressure refolding strategy (Rosa da Silva et al. 2018). The expression of NS1 from DENV-2 in recombinant *E. coli* was obtained as insoluble, and after refolding, the protein was shown to preserve its structural and immunological properties (Amorim et al. 2010; Chura-Chambi et al. 2019). In this context, although refolding of NS1 would be possible, its scale-up would impose additional purification steps, therefore increasing its cost.

In preliminary experiments, we evaluated different strains of *E. coli*, Arctic Express (DE3) and BL21 (DE3), using different temperatures (11 °C and 16 °C) and media (LB, TB and 2xHKSII). Arctic Express (DE3) could support improved folding of heterologous proteins at very low temperatures (4–11 °C) due to two chaperones from the psychrophilic bacterium, *Oleispira Antarctica* (Ferrer et al. 2003). However, our results showed that BL21 (DE3) cultured at 16 °C expressed higher levels of soluble ΔNS1 than Arctic Express (DE3) grown at 11 °C. Of the media evaluated, only TB allowed the production of soluble ΔNS1. The main advantage of TB over LB and 2xHKSII is its buffering capacity, maintaining a controlled pH during growth, and the presence of glycerol as an additional carbon source. TB had previously demonstrated improved performance over other culture media for the production of recombinant proteins (Osadska et al. 2014; Zamani et al. 2015) and plasmid DNA (Wood et al. 2017). The auto-induction medium described by Studier (2005) was also evaluated, but the growth and solubility of ΔNS1 were not better than that observed with TB (data not shown). Thus, the *E. coli* BL21 (DE3) and the TB medium were selected for further optimizations.

We have applied RSM, which allows evaluation of several factors in parallel with a minimum number of experiments, to optimize culture conditions to improve the yield and solubility of ΔNS1 in *E. coli* (Bae and Shoda 2005; Einsfeldt et al. 2011; Emamipour et al. 2019; Uhoraningoga et al. 2018; Xie et al. 2003). From the initial experiments performed using Erlenmeyer flasks, we changed to full-baffled flasks that improve the oxygen transfer rate, approaching the results to that obtained in bioreactors (Tunac 1989). Based on the literature (Berrow et al. 2006; Jia and Jeon 2016), we selected temperature, time and IPTG concentration as the independent variables to evaluate their effect on the responses of biomass concentration and solubility of ΔNS1. Our preliminary data demonstrated that lowering the temperature to 16 °C coupled with induction time of 18 h increased the solubility of ΔNS1 in TB. Therefore, we investigated temperatures from 16 to 30 °C, induction times from 12 to 24 h, and concentrations of the inducer IPTG from 0.4 to 1.0 mM, and included axial points with an alpha of 1.68 in a Central Composite Rotatable Design.

Temperature and time are two of the most important factors affecting the production and solubility of a recombinant protein (Papaneophytou and Kontopidis 2014, 2016). Lowering the temperature can significantly increase the solubility of recombinant proteins (Schein and Noteborn 1988), although this is not always the case and should be evaluated for each protein. Vincentelli et al. (2011) applied high-throughput screening to analyze the effect of *E. coli* strain, culture media and temperature on the solubility of recombinant proteins. Of the 110 prokaryotic proteins tested, induction at 37, 25 and 17 °C resulted in 38, 36 and 25 soluble proteins, respectively. In our statistical model, time presented a significant positive effect for biomass production. On the other hand, temperature had a significant negative effect on the solubility of ΔNS1. The interaction between these factors was also statistically significant with a negative effect on both responses. In the production of soluble recombinant proteins, other studies have also demonstrated that these factors interact with each other (Larentis et al. 2011; Maharjan et al. 2014; Marini et al. 2014; Swalley et al. 2006). Furthermore, in our study, the response surface plots showed a positive curvature and represented well the significance of the quadratic terms of temperature and time of induction, indicating that along with the interaction between these factors, values too high or low will have a negative effect on the responses.

Moreover, the effect of the concentration of IPTG was not significant for any of the responses, but its quadratic term presented a significant negative effect on both biomass formation and ΔNS1 production. IPTG may lead to poor induction or result in inhibitory effects for the cell in a dose-dependent manner (Heyland et al. 2011). In fact, the concentration of the inducer can have a great impact on growth and yield (Einsfeldt et al. 2011; Larentis et al. 2011; Marini et al. 2014) or little to no effect (Ghaderi et al. 2018; Xie et al. 2003). Through statistical designs, Papaneophytou et al. (2013) observed a significant effect of IPTG on the solubility of RANKL, but not for TNF-α (Papaneophytou and Kontopidis 2012), both produced in recombinant *E. coli*. IPTG can also be used in low concentrations when coupled to other inducers such as galactose, in an attempt to decrease the cost and prevent the deleterious effect of IPTG (Restaino et al. 2013). Across all the experiments, biomass concentration varied almost fourfold and soluble ΔNS1 concentration more than 240-fold. This emphasizes the importance of optimizing the culture conditions before scaling-up. If we had applied a standard protocol using lower temperature (induction at 18 °C for 16 h with 0.5 mM of IPTG) (Structural Genomics et al. 2008), we would have obtained soluble ΔNS1, but according to our model at a yield approx. 40% lower. The best conditions predicted by the model were tested and validated. From the predicted responses of 7.5 g/L DCW and 962 mg/L of ΔNS1 we obtained an average of 7.25 g/L DCW and 1022 mg/L ΔNS1, a difference of only 3 and 6%, between predicted and actual values, respectively.

Using the condition established by the RSM (induction at 21 °C with 0.7 mM IPTG) we scaled-up the cultivation to a 6-L bioreactor batch. In comparison to the small-scale flasks, the optimal duration of induction in the bioreactor was lower. These differences may be due to control of dissolved oxygen and pH during the whole culture. Additionally, even with the use of full-baffled flasks, some differences in the growth rate are expected when scaling-up from 100 mL to a 6-L batch. During the batch cultivation in the bioreactor, we observed mainly the addition of phosphoric acid (not ammonium hydroxide), suggesting a tendency towards alkalization. This was previously observed by others and implies a rapid consumption of glycerol as carbon source during the culture (Kram and Finkel 2015; Losen et al. 2004). In this scenario, further improvements could be possible by optimizing the input of the carbon source in a pH-controlled environment, which could be achieved in a batch or fed-batch process. The addition of ethanol up to 2% v/v has been shown to increase the solubility of recombinant catalase expressed in *E. coli* (Zheng et al. 2019) and could be used to further increase the yield. Nonetheless, the current culture conditions allowed production of an estimated 0.5 g/L of ΔNS1 in a 6-L batch culture. Based the current purification setup, with an estimated 20% of ΔNS1 recovery, a single batch would be sufficient for ~ 30,000 ELISA plates.

Despite the broad utilization of the NS1 proteins for flaviviruses serological diagnosis, it has been demonstrated that DENV NS1 proteins lead to conflicting results in endemic areas, especially for ZIKV (Felix et al. 2017; Matheus et al. 2016). Although ZIKV NS1 proteins have been considered specific for ZIKV infections (Matheus et al. 2016), it’s cross-reactivity with DENV is widely acknowledged by the scientific community. Our previous results had shown that the recombinant ΔNS1 fragment was a useful antigen for the detection of specific IgG antibodies generated upon ZIKV infection (Caires-Junior et al. 2018; Kam et al. 2017; Oliveira et al. 2016). Here, the purified ΔNS1 protein was used to characterize the specificity of the antibodies generated by infection with ZIKV and other flavivirus, including human serum samples of patients infected with DENV. The serological tests carried out with ΔNS1 showed a reduced cross-reactivity of the serum from individuals previously infected with DENV as compared to full length NS1. Such enhancement in the specificity may be ascribed to the reduction of common surface exposed epitopes in NS1 between ZIKV and DENV. Despite the structural similarity between the NS1 protein of ZIKV and other flavivirus, ZIKV NS1 displays a divergent electrostatic potential that may explain the altered binding profile of the antibodies to ΔNS1 (Song et al. 2016). Finally, the lack of detection when using denatured ΔNS1 reinforces the relevance of conformational epitopes in the detection of ZIKV-specific antibodies and the essential requirement of soluble protein production.

The data presented in this study demonstrates the application of statistical design to improve the yield and solubility of ΔNS1 produced by recombinant *E. coli*, its pilot-scale production and specificity for serological diagnosis of ZIKV infection. These results will contribute to the viability of this much needed serological diagnostic of ZIKV infection in the presence of other flavivirus.

## Supplementary information

**Additional file 1: Fig. S1.** Evaluation of culture medium and comparison of Arctic and BL21 E. coli strains producing ΔNS1. **Fig. S2** Effect of antifoam on the production of soluble ΔNS1. **Fig. S3** Normal (%) probability plot of the Studentized residuals. **Fig. S4** Reactivity of ΔNS1 and IgG antibodies from mouse infected with ZIKV and other viruses.

## Data Availability

All data generated or analyzed during this study are included in this published article and in additional file. Please turn to the corresponding author for all other requests.
