# Supplementary material for: Optimization and scale-up production of Zika virus ΔNS1 in Escherichia coli: application of Response Surface Methodology
Source: AMB Express. 2019 Dec 31;10:1. doi: 10.1186/s13568-019-0926-y (PMC6938527; doi:10.1186/s13568-019-0926-y)
Supplement: Supplementary file 1 — Additional file 1: Fig. S1. Evaluation of culture medium and comparison of Arctic and BL21 E. coli strains producing ΔNS1. Fig. S2 Effect of antifoam on the production of soluble ΔNS1. Fig. S3 Normal (%) probability plot of the Studentized residuals. Fig. S4 Reactivity of ΔNS1 and IgG antibodies from mouse infected with ZIKV and other viruses. [file 13568_2019_926_MOESM1_ESM.docx]

**Optimization and scale-up production of Zika virus ΔNS1 in *Escherichia coli*: application of Response Surface Methodology**

AMB Express

Alex Issamu Kanno^1^, Luciana Cezar de Cerqueira Leite^1^, Lennon Ramos Pereira^2^, Mônica Josiane Rodrigues de Jesus^2^, Robert Andreata-Santos^2^, Rúbens Prince dos Santos Alves^2^, Edison Luiz Durigon^3^, Luís Carlos de Souza Ferreira^2^, Viviane Maimoni Gonçalves^1*^

^1^ Laboratório de Desenvolvimento de Vacinas, Instituto Butantan, Av Vital Brasil, 1500, São Paulo, SP, 05503-900 Brazil

^2^ Laboratório de Desenvolvimento de Vacinas, Instituto de Ciências Biomédicas, Universidade de São Paulo, São Paulo, SP, Brazil

^3^ Laboratório de Virologia, Instituto de Ciências Biomédicas, Universidade de São Paulo, São Paulo, SP, Brazil

*** Correspondence:**

Dr. Viviane Maimoni Gonçalves

telephone +55 (11) 2627 9821

e-mail address viviane.goncalves@butantan.gov.br

**Electronic Supplementary material**


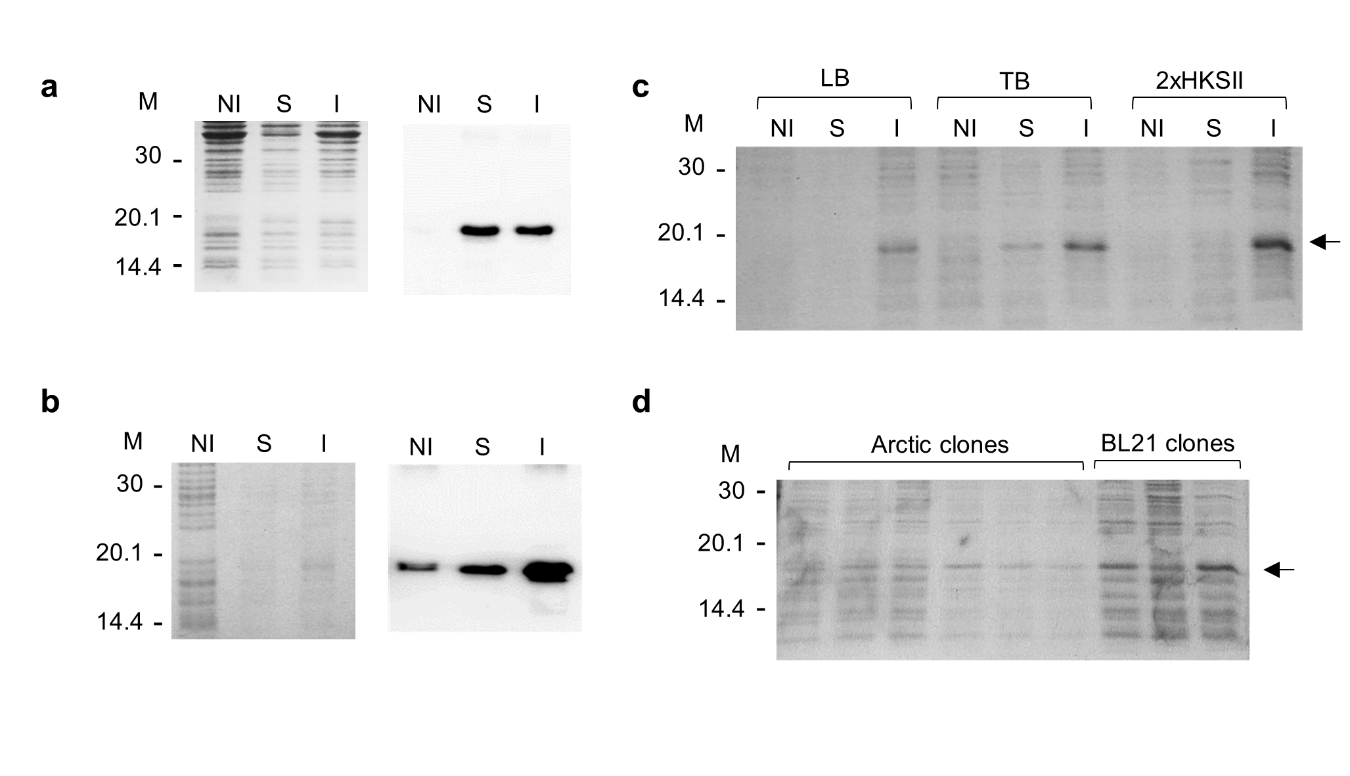


**Fig. S1 Evaluation of culture medium and comparison of Arctic and BL21 E. coli strains producing ΔNS1** Production of ΔNS1 in Arctic strain cultivated in **a** Luria Broth and **b** Terrific Broth. Cultures were induced at an OD ~ 2.0 with 0.5 mM IPTG and kept in shake flasks at 11 ^o^C for 18 h. Protein extracts separated in soluble and insoluble fractions and analyzed in SDS-PAGE and Western blot using anti-his antibody. M = molecular weight marker, NI = non-induced, S = soluble, I = insoluble fraction. **c** SDS-PAGE of protein extracts of BL21 strain producing ΔNS1 (arrow) cultivated in LB, TB and 2xHKSII medium. ΔNS1 was induced as previous and kept at 16 ^o^C for 18 h. **d** Soluble protein extracts (5 µg) of Arctic and BL21 clones producing ΔNS1.


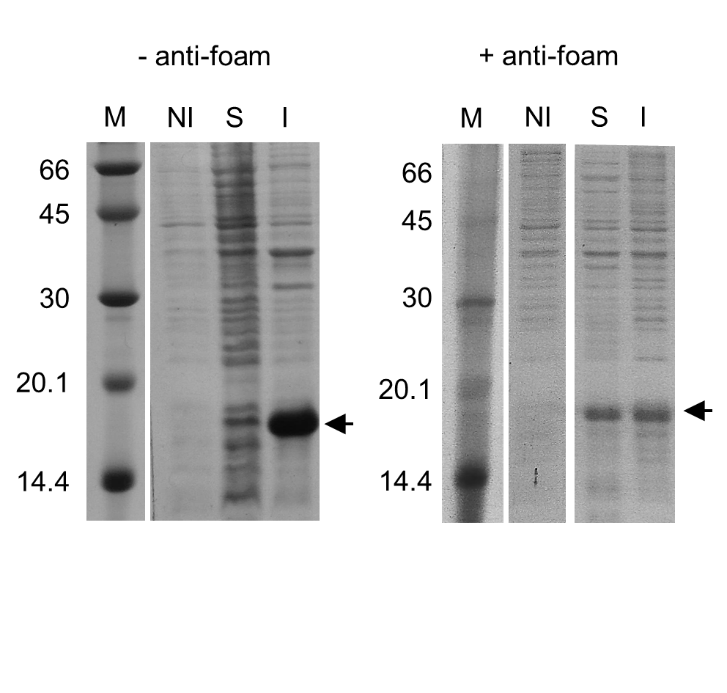


**Fig. S2 Effect of anti-foam on the production of soluble ΔNS1** To produce soluble ΔNS1 using full-baffled TunAir flasks the addition of anti-foam PPG 0.03% was needed. In a 1-L culture (16 ^o^C, 18 h, 0.5 mM IPTG) without anti-foam ΔNS1 (arrows) concentrated in the insoluble fraction. With the addition of anti-foam approximately half of ΔNS1 produced was observed in the soluble fraction.


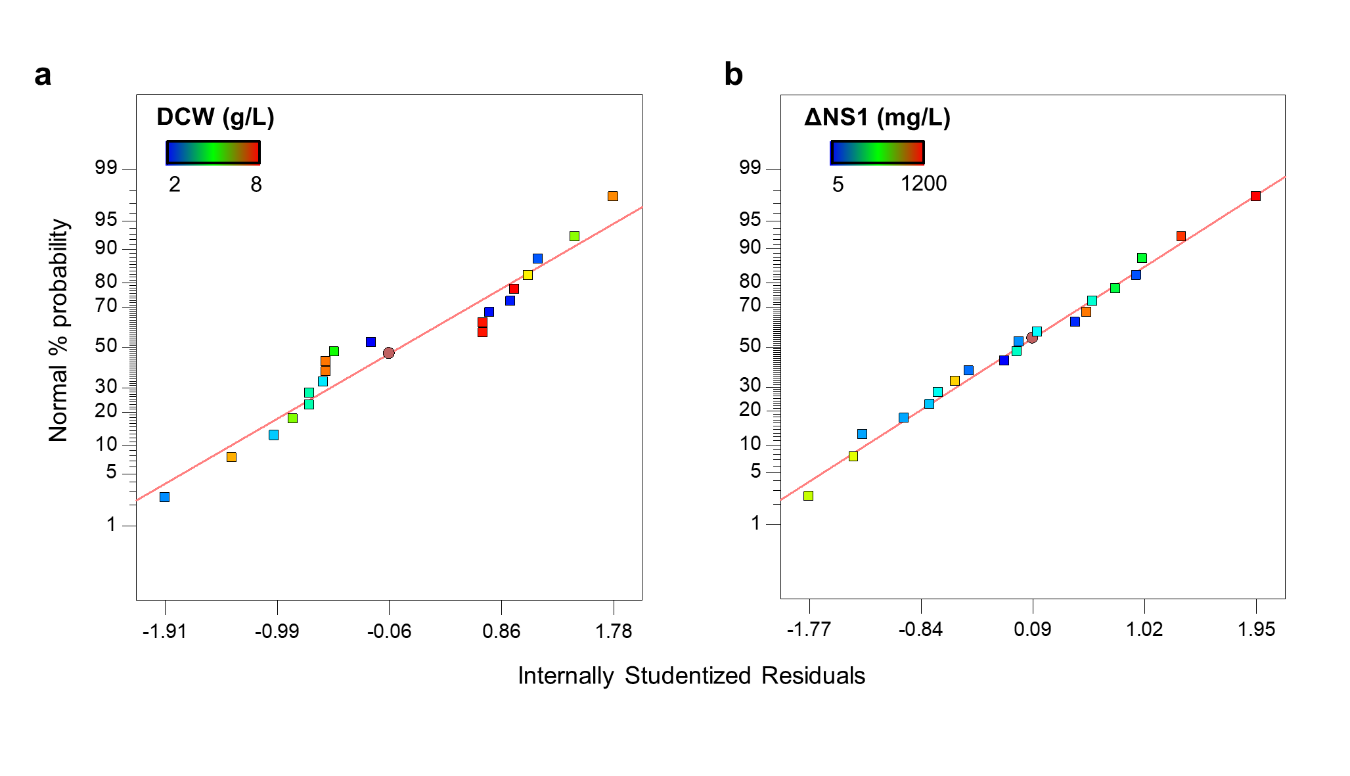


**Fig. S3 Normal (%) probability plot of the Studentized residuals** The normal distribution of the residuals for **a** biomass (DCW) and **b** soluble ΔNS1 production. The residuals (observed minus predicted values) are normally distributed and follow the linear regression (line). Each data point is represented by a color-coded box with its respective value of DCW or soluble ΔNS1. Internally Studentized Residuals (residual divided by the estimated standard deviation of that residual) measures the number of standard deviations separating the actual and predicted values. Centered dot represents the mean standard deviation.


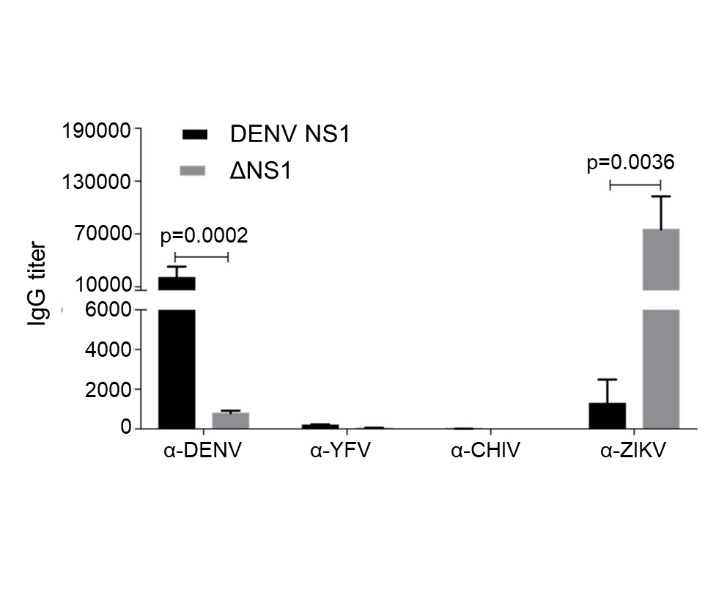


**Fig. S4 Reactivity of ΔNS1 and IgG antibodies from mouse infected with ZIKV and other viruses** The purified proteins DENV2 NS1 and ZIKV ΔNS1 were reacted by ELISA with pooled ascitic fluid from mice infected with DENV, YFV, CHIKV or ZIKV. Statistical significance (p-value) was calculated using two-way ANOVA and the Bonferroni test.
